# Small Duct Primary Sclerosing Cholangitis: An Underdiagnosed Cause of Chronic Liver Disease and Cirrhosis

**DOI:** 10.7759/cureus.7298

**Published:** 2020-03-17

**Authors:** Smit Deliwala, Saira Sundus, Tarek Haykal, Mamoon M Elbedawi, Ghassan Bachuwa

**Affiliations:** 1 Internal Medicine, Hurley Medical Center, Michigan State University College of Human Medicine, Flint, USA; 2 Internal Medicine, Hurley Medical Center, Michigan State University, Flint, USA; 3 Gastroenterology, Hurley Medical Center, Michigan State University, Flint, USA

**Keywords:** small duct psc, psc variant, biliary diseases, end stage liver disease, large duct psc, classic psc, cholangiography, liver transplantation, mrcp, ercp

## Abstract

Classic or large duct primary sclerosing cholangitis (PSC) is part of the PSC spectrum. It is diagnosed on clinical and biochemical findings of cholestasis supported by biliary tree changes on cholangiography, forgoing the need for an invasive liver biopsy. The spectrum contains various PSC variants with distinct clinical courses and outcomes. We present a case of small duct PSC, a rare variant that manifested insidiously with clinical and objective cholestasis but appeared negative on diagnostic cholangiography. Eventually, a liver biopsy was obtained that revealed chronic bilious disease of the small and microscopic ducts with simultaneous changes consistent with liver cirrhosis. Despite presenting like its classical counterpart, small duct PSC can remain undetectable on cholangiography due to the diminutive size of the bile ducts requiring histological confirmation. In contrast to classic PSC, small duct PSC portends a much better prognosis. However, it eventually progresses to the classic form or end-stage liver disease, requiring patients to receive timely surveillance and transplantation referrals. Due to the limited understanding of this disease process, patients with similar presentations often pose a diagnostic dilemma due to the clinical and cholangiographic mismatch. This case aims to reaffirm that a negative cholangiography does not rule out the PSC spectrum and that small duct disease is a rare but growing entity. The paucity in cases emphasizes the importance of isolated reports in guiding workup and management, especially since surveillance schedules and transplantation guidelines have not been formally established.

## Introduction

Classic primary sclerosing cholangitis (PSC) or large duct PSC is a well-established entity characterized by its chronic progressive inflammation, fibrosis, and stricturing of large bile ducts along the entire biliary tree. Despite its validation by various long-term follow-up studies demonstrating a median transplantation-free survival of 12 years from diagnosis, it carries a three-fold higher mortality rate and the likelihood of adverse health outcomes than the general population [1-3]. Classic PSC comes under the PSC spectrum, which includes two rare variants, small duct PSC and an overlap syndrome with autoimmune hepatitis; both of these entities have their unique characterizations and require tissue diagnosis for confirmation, unlike the classic form [4-6]. Clinical cases of cholestasis that reflect PSC on chemistry and histology, but appear normal on diagnostic cholangiography are termed small duct PSC (sometimes referred to as pericholangitis). We report a rare case of small duct PSC, a variant that can affect the entire biliary system from the interlobular bile ducts to the ampulla of Vater with its distinctive clinical course and outcome rates [7]. In an age- and sex-adjusted study, the incidence of PSC was 0.9 per 100,000 person-years and twice as significant in men, while small duct PSC was seen in 0.15 per 100,00 person-years [8,9]. Small duct PSC was first described at Mayo Clinic in 1985 and incorporated into the PSC spectrum despite its radiological mismatch [10]. This mismatch is often due to the sheer fact that ducts can be as small as 100 μm in diameter and only be visible via biopsy. Its prevalence is usually higher within younger ages than older adults, and early diagnosis of PSC is more likely to reflect small duct PSC than a late diagnosis [11]. Fifty percent of patients have advanced fibrosis (metavir stage 3 or 4), while 37.5% progress to large duct disease within a median time frame of six years after diagnosis [12]. Prompt recognition is vital as small duct PSC is known to respond better to standard therapy than the classic form [13]. Hence, in patients with features of PSC on laboratory workup and negative cholangiographic evaluation, a biopsy may help rule out PSC variants. The presentation of small duct PSC with decompensated cirrhosis at the time of presentation is incredibly rare, with two similar cases reported in the literature, and a few isolated reports of patients from long-term follow-up studies. This case aims to highlight the clinical nature of small duct PSC and the role of biopsy in the PSC spectrum. In contrast to the well-characterized classic PSC, the scarcity of reported small duct PSC cases often makes diagnosis and management arbitrary, giving precedence to isolated reports to guide medical decision making.

## Case presentation

**Figure 1:**
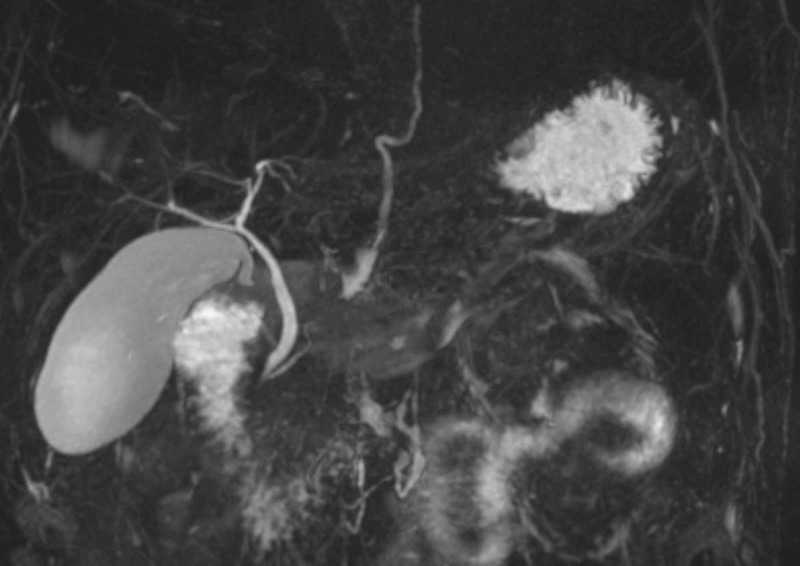
Initial magnetic resonance cholangiopancreatography (MRCP) during the outpatient visit

A 49-year-old male established at our clinic had been experiencing diarrhea and abdominal discomfort not amenable to any over-the-counter antidiarrheals for months. His medical history was significant for diabetes, hypertension, chronic kidney disease, latent syphilis, avascular necrosis of his right hip, and reflux disease. He was known to have chronic transaminitis at baseline and recurrent episodes of jaundice that resolved spontaneously, while the previous workup for hepatitis and mineral overload disorders were negative. At an earlier visit, a magnetic resonance cholangiopancreatography (MRCP) was unrevealing for any hepatobiliary abnormalities apart from sludge (Figure 1). His episodes of jaundice and diarrhea (often unprovoked) would dissipate within 24-48 hours and were often self-managed, relegating any diagnostic workups or hospital visits. Unlike previous events, his jaundice would not decrease, and he characterized his diarrhea as persistent, with over 10 to 20 mucus-like bowel movements a day associated with tenesmus and abdominal cramping. He denied any changes in lifestyle patterns or weight and ate mostly home-cooked meals. He denied ever using tobacco products, alcohol, or illicit substances, nor was he overweight. He came back for an unscheduled clinic visit visibly jaundiced, endorsing abdominal pain, pruritus, dark-colored urine, and pale stools that began spontaneously over the preceding days. Outpatient labs were revealing for anemia, kidney injury, metabolic acidosis, hypoalbuminemia, and high cholestatic markers. He was requested to be admitted to the hospital urgently for further workup and consultation with gastroenterology with a working diagnosis of primary sclerosing cholangitis or a pancreatic pathology.

**Figure 2:**
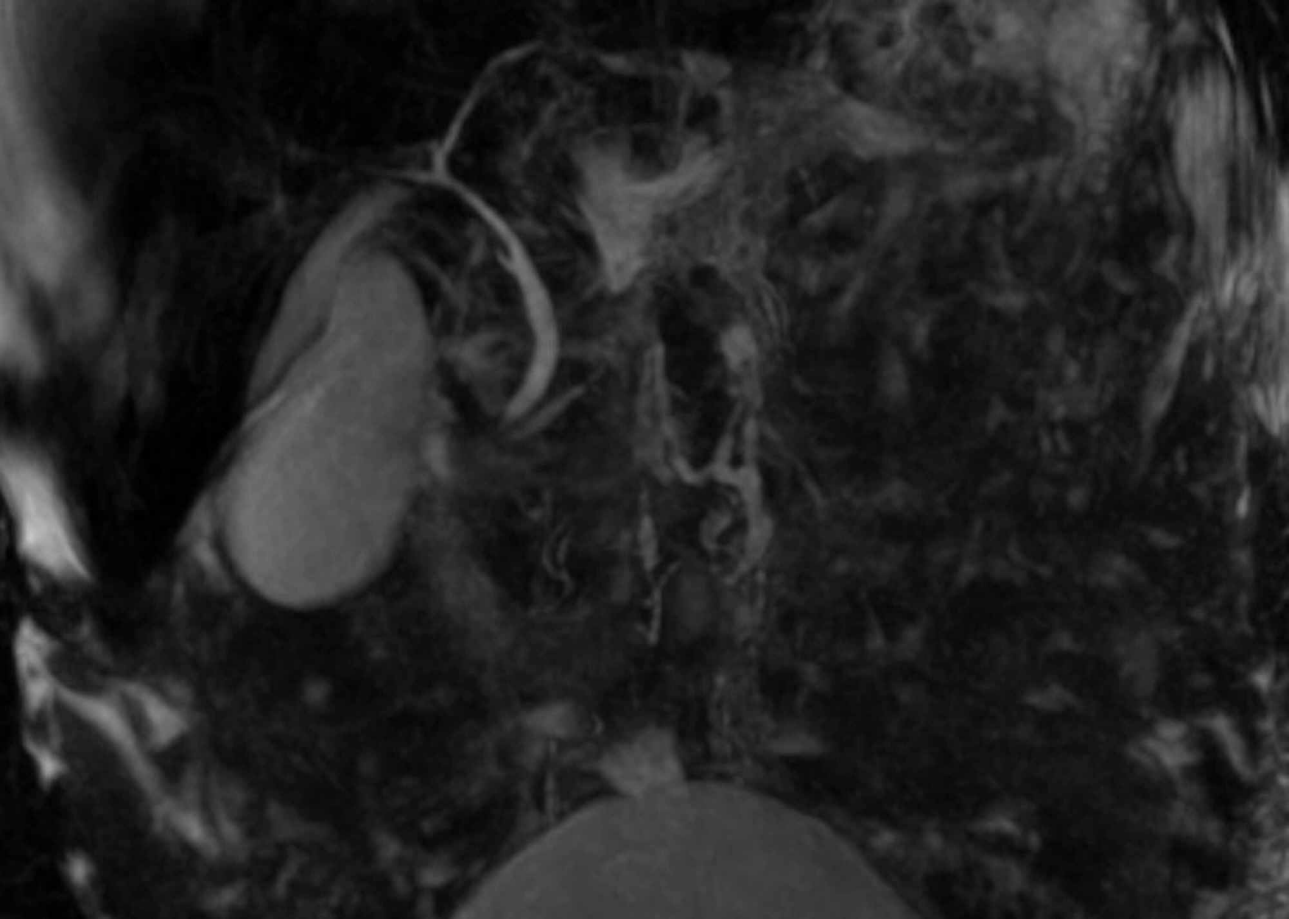
Repeat magnetic resonance cholangiopancreatography (MRCP) during the inpatient workup

**Figure 3:**
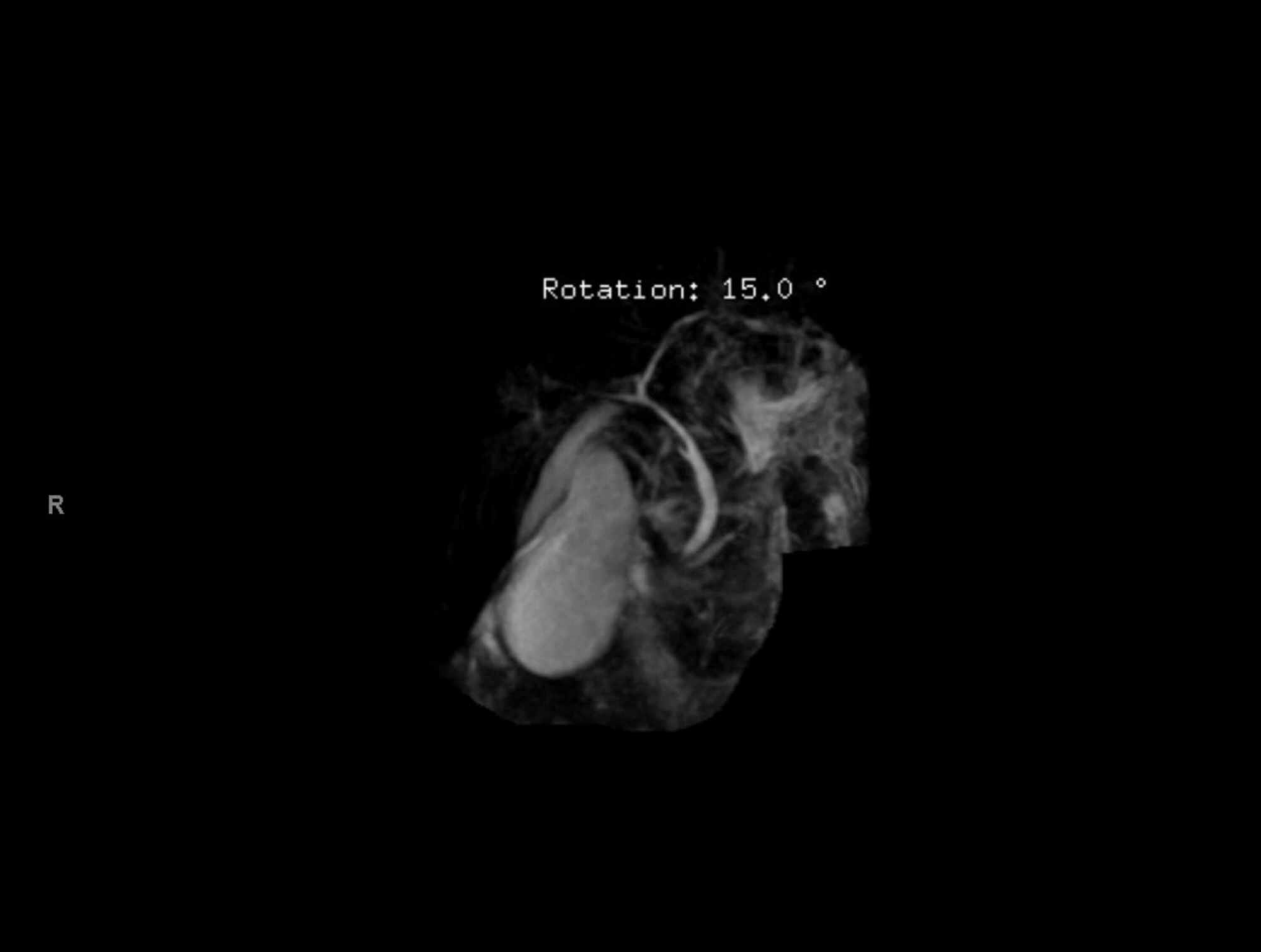
Repeat magnetic resonance cholangiopancreatography (MRCP) during the inpatient workup

An inpatient MRCP (Figure 2) revealed a large amount of biliary sludge, distended gallbladder, and pericholecystic fluid without evidence of common bile duct (CBD) dilatation or stones (Figure 3). Early into his hospitalization, lack of white cell count or inflammatory marker elevation with negative biliary tree changes, ruled out acute cholangitis while a lipase of 30 U/L ruled out pancreatitis. Autoimmune labs were non-contributory as well (Table 1). His abdominal distension was ascitic, and a subsequent paracentesis was unremarkable. Upon completion of a liver workup, a liver biopsy was pursued due to his rapidly declining clinical status and negative MRCP. The liver biopsy demonstrated cirrhosis on trichrome staining with irregularly shaped garland-like nodules representing chronic biliary disease with marked canalicular cholestasis and ductular reactions in the periportal areas. Surveillance colonoscopy was unrevealing for any mucosal changes. During this time, his bilirubin continued to increase, and he developed signs of anasarca. His liver biopsy was read as cirrhosis, most likely due to chronic cholestasis. No targetable lesions, including strictures or dilatations, were noted on MRCP forgoing an endoscopic retrograde cholangiopancreatography (ERCP).

**Table 1:** Small duct primary sclerosing cholangitis (PSC) investigations

Laboratory test	Value
Hemoglobin	8.5 g/dL
White blood cell	6.3 K/uL
Platelet count	197 K/uL
Haptoglobin	302 mg/dL
Lactate dehydrogenase (LDH)	205 U/L
Reticulocyte absolute count	0.16 M/uL
Creatinine	2 mg/dL
Alkaline phosphatase	1013 U/L
Aspartate aminotransferase (AST)	98 U/L
Alanine aminotransferase (ALT)	78 U/L
Total bilirubin	9.4 mg/dL
Bilirubin Indirect	1.5 mg/dL
Bilirubin direct	6.5 mg/dL
Albumin	2.9 gm/dL
International normalized ratio (INR)	1.5
Lipase	30 U/L
Ammonia	51 umol/L
Erythrocyte sedimentation rate (ESR)	>120 mm/hr
HIV 4G Ag/Ab	Negative
Liver-Kidney Microsomal antibody	0.7 U
Smooth muscle (F-actin) IgG antibody	15 U
Mitochondrial antibody	Negative
Cancer antigen (CA) 19-9	49.5 U/mL
Carcinoembryonic antigen (CEA)	1.2 ng/mL
Immunoglobulin (IgG4)	59 mg/dL
Alpha-fetoprotein CA	2.7 ng/mL
Iron saturation	35%
Hepatitis B surface antibody	Negative
Hepatitis B surface antigen	Negative
Hepatitis C antibody	Negative
Tissue transglutaminase	14 U
Anti-nuclear antibody (ANA)	1:160 in a speckled pattern
P-ANCA	<1:20 Titer
C-ANCA	<1:20 Titer

Home medications were restarted, and intravenous fluids were given for dehydration and kidney injury. He was started on ursodeoxycholic acid and hydroxyzine for pruritis and loperamide for diarrheal relief. Shortly after his biopsy resulted, our primary working diagnosis was PSC despite the cholangiographic features. Once cirrhosis was evident from his biopsy, we calculated his Model For End-Stage Liver Disease (MELD) score to 32, and with simultaneous worsening of his disease, we transferred him to a tertiary center with access to advanced gastroenterology services for transplant candidacy. At the tertiary center, he was formally diagnosed with small duct PSC by a hepatologist based on his biopsy specimen and clinical status forgoing an ERCP. Esophagogastroduodenoscopy (EGD) and repeat colonoscopy and its biopsies were unremarkable.

## Discussion

The natural disease progression of small duct PSC has primarily remained unexplored with only a handful of long-term studies on small duct PSC, mostly from Scandinavian and American models [14,15-17]. From an epidemiological standpoint, large duct PSC is often more prevalent among women, while the incidence of small duct PSC has been increasing significantly among men. However, age was shown to have no significant effect on the incidence, contrary to historical studies that conferred a better prognosis for diagnoses at a younger age [18]. Long-term prognostic data for small duct PSC compared to classic PSC is not robust; however, the three most extensive follow-up studies were able to uniformly conclude that small duct PSC had a better long-term prognosis compared to large duct PSC [15-17]. These studies demonstrated lower numbers of deaths, liver-related deaths, liver fibrosis, and liver transplantation rates compared to the large duct group [1,15]. The longest of these studies had a median follow-up of 29.5 years, and patients who remained asymptomatic had better outcomes than their symptomatic counterparts [17]. These findings suggest that small duct PSC represents a set of patients within the sclerosing cholangitis spectrum with a favorable long-term prognosis compared to those with cholangiography-proven PSC [19].

Approximately 12%-25% of patients with small duct PSC progressed to large duct disease with an average follow-up time of eight years from the first ERCP [16]. In patients who progressed to large duct disease, 47% either died or underwent a transplant, while progression to large duct disease significantly increased the risk of progression to end-stage liver disease or development of cholangiocarcinoma (CCA), postulating that small duct PSC must be a precursor to large duct PSC. Interestingly, a small subset of patients who underwent OLT developed recurrent small duct PSC in the graft requiring re-transplantation, which heralds a concerning feature of its ability to recur even after liver transplant, similarly as autoimmune pathologies [1,15]. The use of ursodeoxycholic acid at 13-15 mg/kg/day was associated with biochemical improvements similar to large duct PSC but did not appear to prevent the progression of the disease [20].

Liver transplantation is an essential step as 17% of patients with small duct PSC with large duct progression eventually progress to end-stage liver disease, and 55% of patients dying from liver-related deaths [1]. Histological specimens often reveal mild inflammation in the majority of the cases with onion ring appearance on biopsy specimens [1,19]. Liver biopsy continues to be a critical diagnostic tool in patients with small duct PSC. Patients who did not develop large duct disease often still developed adverse outcomes from liver-related complications, mirroring our patient's clinical course [1,15]. The most feared complication of classic PSC largely remains the development of CCA. In a study of 32 Swedish patients, none developed cholangiocarcinoma while PSC patients have a lifetime risk of 10%-20% [16,17]. Small duct PSC patients also demonstrated longer median survival-free time from liver transplantation [1]. Although OLT has supplanted many adverse outcomes in PSC, rates of CCA have largely remained unchanged, while rates of CCA originating from small duct PSC are almost non-existent [5]. Interestingly enough, no cases of CCA were seen unless there was progression to large duct disease first. This sets the premise of CCA being an offshoot of large ducts than small and these findings support the hypothesis that large duct epithelium is required for CCA development, while small duct epithelium seems to confer protection to the carcinogenicity [1].

Classic PSC has a strong association with inflammatory bowel disease (IBD), specifically ulcerative colitis, with rates approaching 70% [3]. In contrast, only 5% of patients with IBD have PSC and routine screening is not always employed in these cases [4]. Small duct PSC patients share the same risk of developing colonic dysplasias as classic PSC patients [19]. Furthermore, patients with PSC-IBD demonstrate a phenotypic difference than patients with IBD alone. Likewise, undifferentiated colectomy rates for IBD did not differ between small duct PSC and large duct PSC and the development of IBD had no impact on liver-related morbidity and mortality either. The prevalence of IBD among the two groups seems to present similarly, while Crohn's disease was noted to be higher in small duct PSC in a study of 305 patients at nearly 7%-21%, much higher than previously referenced data, with a median follow-up time of 13 years [1]. The association with IBD does not appear to alter the course of liver damage.

Small duct PSC is a diagnosis of exclusion supplemented by abnormal liver function tests, normal cholangiogram, and a liver biopsy consistent with PSC morphology. A negative cholangiographic evaluation does not rule out the PSC spectrum as variants can only be identified on tissue biopsy unlike classic PSC, that can be diagnosed on cholangiographic grounds. At our institution and at the tertiary facility, it was felt that an ERCP would not serve any further purpose as a negative cholangiogram strongly rules out PSC with a meta-analysis of six studies revealing a sensitivity and specificity of 86 and 94 percent respectively, while ERCP is often reserved for patients that cannot tolerate MRCP, management of strictures, acquisition of cytology or if conversion to classic PSC occurs [14]. In a large study of patients with small duct in the pre-ERCP era, all patients were diagnosed by means of cholangiograms and biopsies [7].

## Conclusions

Small duct PSC is a diagnosis of exclusion, considered in patients presenting with chronic cholestasis and a negative cholangiography. Despite a progressive cholestatic course, it carries a more favorable prognosis than classic PSC. Although formal recommendations have not been established, patients require routine surveillance, and transplant evaluation if significant liver disease progression has been demonstrated. Cirrhosis has been a well-known complication of small duct PSC, although reports are far and few making this a notable aspect of our case. It serves as a learning lesson that a negative cholangiogram does not rule out the PSC spectrum and that the natural progression of this disease can be devastating if not suspected early. MRCP, ERCP or percutaneous transhepatic cholangiography (PTC) are available modalities for workup, but tissue diagnosis remains the only way to confirm the PSC spectrum and its variants. Laboratory markers such as serum bilirubin level impact on long-term survival within the small duct cohort have not been elucidated, a potential avenue for future research while short- and long-term outcome data are limited, with scarce isolated reports to guide management. Often the most dreaded complication of the PSC spectrum is CCA, although, within small duct PSC, the conversation rate is minimal to non-existent. Certain authors maintain that because of this finding, CCA may be a disease of large ducts, although further research is needed on this matter.
